# Sarcoidosis exosomes stimulate monocytes to produce pro-inflammatory cytokines and CCL2

**DOI:** 10.1038/s41598-020-72067-7

**Published:** 2020-09-18

**Authors:** Casper J. E. Wahlund, Gozde Gucluler Akpinar, Loïc Steiner, Ahmed Ibrahim, Elga Bandeira, Rico Lepzien, Ana Lukic, Anna Smed-Sörensen, Susanna Kullberg, Anders Eklund, Johan Grunewald, Susanne Gabrielsson

**Affiliations:** 1 Division of Immunology and Allergy, Department of Medicine Solna, Karolinska Institutet, Solna, Sweden; 2 Department of Clinical Immunology and Transfusion Medicine, Karolinska University Hospital, Solna, Sweden; 3 Respiratory Medicine Division, Department of Medicine Solna, Karolinska Institutet, Solna, Sweden; 4 Department of Respiratory Medicine, Karolinska University Hospital, Solna, Sweden; 5 Department of Clinical Neuroscience, Therapeutic Immune Design, Karolinska Institutet, Solna, Sweden

**Keywords:** Chronic inflammation, Respiratory tract diseases

## Abstract

Pulmonary sarcoidosis has unknown etiology, a difficult diagnostic procedure and no curative treatment. Extracellular vesicles including exosomes are nano-sized entities released from all cell types. Previous studies of exosomes from bronchoalveolar lavage fluid (BALF) of sarcoidosis patients have revealed pro-inflammatory components and abilities, but cell sources and mechanisms have not been identified. In the current study, we found that BALF exosomes from sarcoidosis patients, but not from healthy individuals, induced a dose-dependent elevation of intracellular IL-1β in monocytes. Analyses of supernatants showed that patient exosomes also induced release of IL-1β, IL-6 and TNF from both PBMCs and enriched monocytes, suggesting that the observed effect is direct on monocytes. The potently chemotactic chemokine CCL2 was induced by exosomes from a subgroup of patients, and in a blocking assay the exosome-induced CCL2 was reduced for 13 out of 19 patients by the asthma drug Montelukast, a cysteinyl leukotriene receptor antagonist. Further, reactive oxygen species generation by PBMCs was induced to a higher degree by patient exosomes compared to healthy exosomes. These findings add to an emerging picture of exosomes as mediators and disseminators of inflammation, and open for further investigations of the link between CCL2 and exosomal leukotrienes in sarcoidosis.

## Introduction

Sarcoidosis is an enigmatic disorder of unknown cause, potentially affecting any organ of the body, with pulmonary involvement in approximately 90% of all patients^1^. The main pathological finding is granulomas, complex formations of fusing epithelioid cells and immune cells including CD8^+^ and CD4^+^ T cells, with inflammatory cytokines such as IFNγ and TNF having central roles^2^. Exosomes are nano-sized membrane vesicles capable of transporting a diverse set of nucleic acids, proteins and lipids^3^. Extracellular vesicles (EVs) include exosomes, which are of endosomal origin. We here refer to small EVs isolated by differential centrifugations as exosomes, as in our previous studies^4,5^.

We previously found that BALF exosomes from sarcoidosis patients are enriched in CD54 and MHC molecules and induce IFNγ in autologous PBMCs, presumably contributing to inflammation^4^. We have further proposed that EVs from the lungs may be vehicles initiating or aggravating inflammatory events, both in pulmonary compartments as well as systemically or in distant organs^6^. In line with this, we hypothesise that EVs play a role in augmenting sarcoid inflammation. Studies on cellular players in sarcoidosis include both innate and adaptive immune cells, however with a strong emphasis on T cells, which are considered drivers of the disease^2^. We have seen that sarcoidosis BALF exosomes have a shift in the leukotriene profile and are enriched for a large number of pro-inflammatory components, several of which have chemotactic properties on T cells, but also on monocytes^5^. The mononuclear phagocyte (MNP) system comprises cell types of greatly overlapping phenotype and function. Its implication in pulmonary disorders, including sarcoidosis, is of increasing interest^7,8^, and tissue infiltration of MNPs is a key hallmark of sarcoidosis^9^. Peripheral blood monocytes in sarcoidosis patients display a skewing towards intermediate (CD14^+^CD16^+^) monocytes, which are associated with inflammatory conditions^10–12^. Further, sarcoidosis monocytes have been found to have a decrease in expression of CD200R, a phenotype associated with inflammatory disease^11^. Moreover, during inflammation in general, monocytes are recruited from peripheral blood to affected tissues and modulate inflammatory processes, in part by releasing large amounts of cytokines^13^. TNF, which is readily produced by stimulated monocytes, is a key cytokine in sarcoid granulomas, and anti-TNF immunotherapy is deployed as treatment regimen for some sarcoidosis patients^14,15^.

Taken together, it is likely that monocytes play a role in sarcoidosis, but it is unclear via which mechanisms and to what degree they may affect sarcoid inflammation. Beyond pro inflammatory cytokines and chemokines^1,2^, sarcoidosis patients show elevated oxidative stress including increased reactive oxygen species (ROS)^16^, which monocytes produce in inflammatory situations^17–19^. We therefore investigated whether PBMCs and monocytes can be engaged by sarcoidosis exosomes to favor an inflammatory profile in PBMCs and/or monocytes. Exosomes induced a dose-dependent intracellular IL-1β increase in classical (CD14^+^CD16^−^) monocytes amongst PBMCs, but also in enriched monocytes suggesting a direct effect. Patient exosomes stimulated the release of IL-1β, IL-6 and TNF from both PBMCs and enriched monocytes. Further, for 22 h stimulated PBMCs the levels of CCL2 could be reduced in 13 out of 19 patient exosome stimulations using Montelukast, a commonly used asthma drug. In a separate validation cohort, the blocking assay was repeated and showed that Montelukast decreased CCL2 levels for approximately half of the 30 patient exosome stimulations. This opens for further investigations of exosome effects on sarcoid inflammation, and of Montelukast as an inhibitor of exosome-mediated inflammation in certain sarcoidosis patients.

## Results

### In sarcoidosis, the BALF exosome numbers are elevated

**Figure 1:**
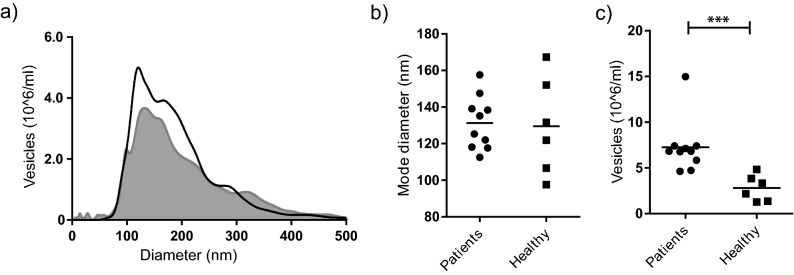
BALF exosome numbers are elevated in sarcoidosis patients compared to healthy individuals. BALF exosomes from ten patients with sarcoidosis and six healthy volunteers were analysed using nanoparticle tracking analysis. (**A**) Size distributions were similar for patient exosomes (open histogram) and healthy exosomes (grey closed histogram). The exosome mode diameters (**B**) did not differ between the groups, but patient BALF had approximately twice the number of exosomes compared to healthy BALF (**C**).

Using nanoparticle tracking analysis, we found that the ultracentrifuged preparations from BALF of sarcoidosis patients and healthy volunteers contained exosomes of similar size distributions (Fig. 1A) and mode diameters (131 nm and 130 nm for patients and healthy controls respectively, Fig. 1B). Corroborating our earlier findings where we found more exosomal protein in patient compared to healthy control BALF^4^, exosomes were more abundant in the BALF from patients (average 7.3 × 10^6^ vesicles/ml) compared to healthy controls (2.8 × 10^6^ vesicles/ml, Fig. 1C).

### Sarcoidosis exosomes dose-dependently elevate IL-1β in classical monocytes

**Figure 2:**
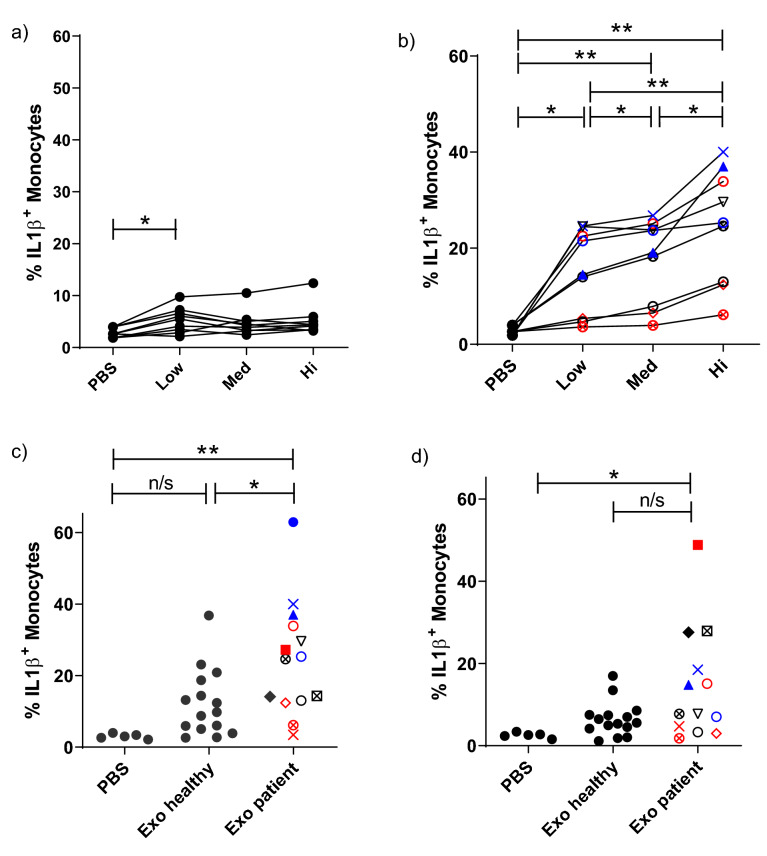
Sarcoidosis BALF exosomes induce a dose-dependent IL-1β increase in classical monocytes. Healthy donor PBMCs were stimulated for 6 h with exosomes from 1, 5 or 15 ml (Low, Med, Hi) of BALF from healthy volunteers (**A**) or sarcoidosis patients (**B**). PBS equivalent to the exosome volume was used as negative control. After 6 h, the cells were washed and stained for flow cytometric analysis. Classical monocytes were gated as single, lineage^−^ (CD3, CD19, CD56, CD66b), HLA-DRhi, CD14^+^CD16^−^ cells (gating strategy in Supplementary Fig. 1A). The proportions of classical monocytes positive for intracellular IL-1β are displayed (typical plots in Supplementary Fig. 2). To investigate if the observed effect was direct on monocytes or mediated via other cells, both PBMCs (**C**) and enriched monocytes (**D**) were stimulated in parallel with exosomes from 15 ml of BALF. An equivalent volume of PBS was used as negative control. n = 9 healthy exosome donors and 9 sarcoidosis patients were distributed to recipient cells from three healthy donors in (**A**) and (**B**). n = 13–14 patient exosomes and 15 healthy donor exosomes were distributed to recipient cells from five different healthy volunteers in (**C**) and (**D**). A repeated measure one way Anova with Tukey’s post-hoc test was used in (**A**) and (**B**), an ordinary one way Anova test with Tukey´s post hoc test was used in (**C**) and (**D**). *p < 0.05, **p < 0.01.

We have previously detected cytokine responses to sarcoidosis exosomes in autologous cell stimulations^4^. To further investigate the inflammatory capacity of patient exosomes with focus on innate responses, we added BALF exosomes from sarcoidosis patients or healthy individuals to allogeneic PBMCs and analysed the induction of IFNγ and IL-1β in different cell subsets. PBMCs from healthy donors (500,000 per well) were stimulated with exosomes isolated from 1, 5 or 15 ml BALF of sarcoidosis patients and analysed by intracellular flow cytometry. Gating strategies are displayed in Supplementary Fig. 1A,B, and typical IL-1β findings in Supplementary Fig. 2. After 6 h stimulations with exosomes, neither T cells (CD4^+^ and CD8^+^, respectively) nor NK cells showed any increase in intracellular IFNγ (Supplementary Fig. 3). However, patient exosomes induced IL-1β expression in monocytes: healthy donor exosomes showed a minimal effect (Fig. 2A), but patient exosomes induced a significant, dose-dependent increase in intracellular IL-1β in (classical) monocytes (Fig. 2B).

**Figure 3:**
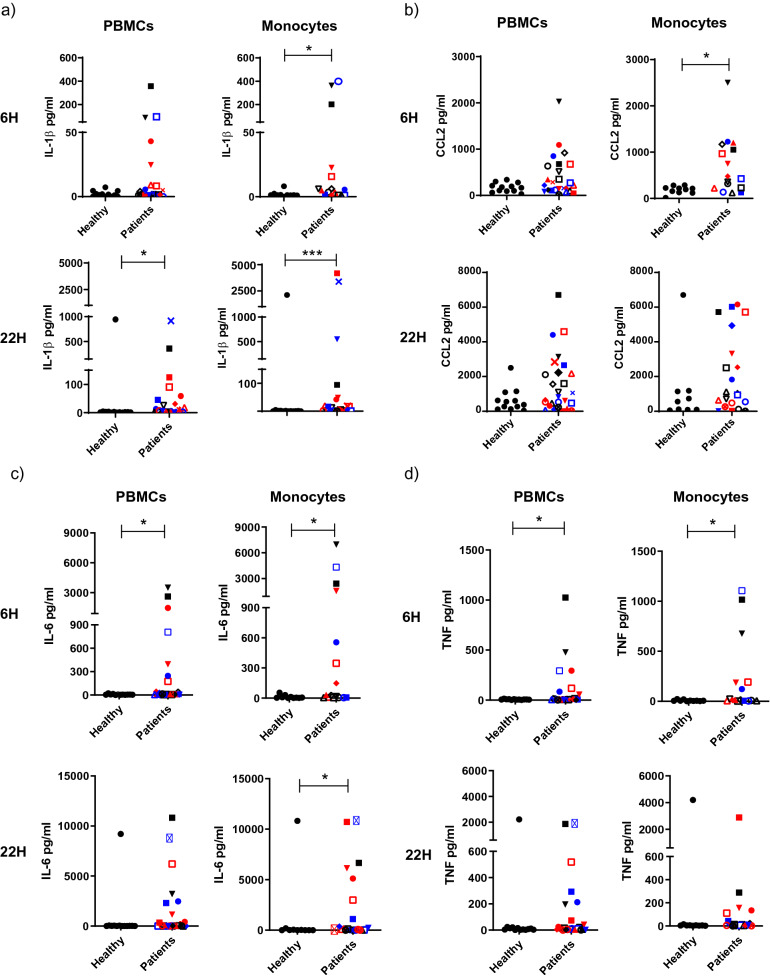
Sarcoidosis exosomes induce pro-inflammatory cytokine release from both PBMCs and enriched monocytes. PBMCs or enriched monocytes from healthy donors were stimulated for 6 or 22 h with exosomes from 5 ml BALF from sarcoidosis patients or healthy donors. PBS was used as negative control. A multiplex cytokine assay acquired by flow cytometry was used to analyse concentrations of IL-1β (**A**), CCL2 (**B**), IL-6 (**C**), and TNF (**D**) in supernatants. *p < 0.05, **p < 0.01, ***p < 0.001 using a Wilcoxon test. n = 19–27 patient exosomes and 10–13 healthy volunteer exosomes distributed on at least four healthy recipient cell donors.

To investigate whether this effect on monocytes was direct or indirect, both PBMCs and enriched monocytes from the same healthy donors were stimulated in parallel. IL-1β was again increased in classical monocytes within the PBMC population stimulated with patient exosomes compared to healthy exosomes or PBS (Fig. 2C). When stimulating the monocyte-enriched population (75% CD14^+^ cells, Supplementary Fig. 1A) with exosomes, similar effects on IL-1β production in monocytes were seen as in the PBMC stimulations (Fig. 3D).

### Sarcoidosis exosomes induce release of IL-1β, TNF, IL-6 and CCL2

To relate the findings of elevated IL-1β^+^ monocytes with released cytokines, a multiplex cytokine assay was performed on supernatants of PBMCs or enriched monocytes which were stimulated with exosomes for six or 22 h. No significant levels of released IFNγ were seen (data not shown), but IL-1β release was significantly induced in enriched monocytes at 6 h, and in both PBMCs and enriched monocytes at 22 h (Fig. 3A). The strongly chemotactic CCL2 was induced by exosomes from a subgroup of patients (7–8 out of 20–28 depending on time point and cell type), showing statistical significance for enriched monocytes at 6 h (Fig. 3B). Further, both IL-6 and TNF were significantly induced by patient exosomes at 6 h for both PBMCs and enriched monocytes (Fig. 3C, D; upper graphs). After 22 h, significance was only seen in stimulated enriched monocytes for IL-6 secretion (Fig. 3C, D lower graphs). To elucidate if high inducing exosomes were derived from patients sharing any common clinical characteristics, we correlated the findings to substantial cell- and clinical data: BAL cell numbers, cell concentrations, differential counts, disease staging, age, gender, smoking habits, lung capacity measured in FVC, FEV1 and DLCO, as well as blood cell concentrations and medication, however without finding any clear correlation (data not shown). The patient exosomes inducing the higher levels of released cytokines were also compared to those inducing the higher IL-1β induction in monocytes (Fig. 2), again with no apparent pattern for these patients (data not shown). In addition, in general, it was not the same patients that were high inducers for the different cytokines. In initial studies in four patients and four healthy volunteers, we analysed other cytokines associated to a broad repertoire of cells (including T cells) and functions; GM-CSF, IL-12p70, IL-2, IL-5, IL-10 and IL-17A. These were however not induced to detectable levels by any exosomes (data not shown).

### Montelukast reduces sarcoidosis exosome-induced CCL2 production in a subgroup of patients

**Figure 4:**
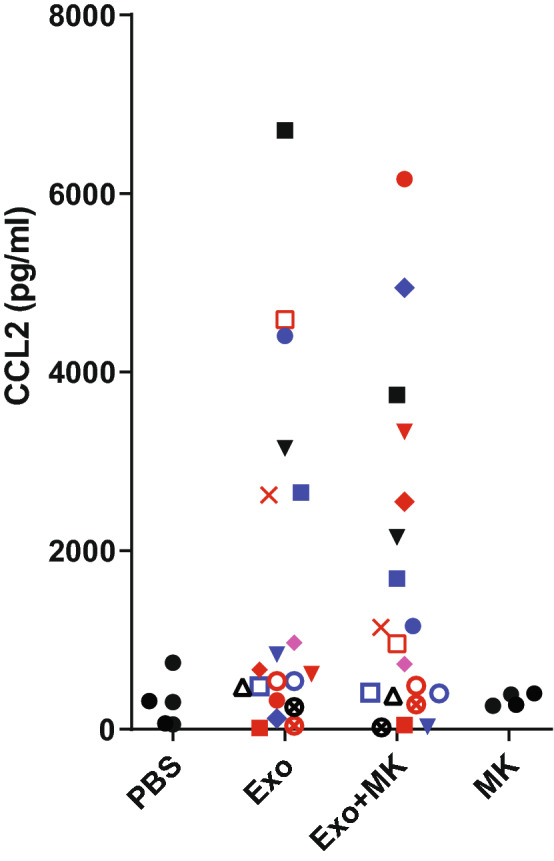
Montelukast reduced CCL2 levels for a subgroup of patient exosome stimulations. Healthy donor PBMCs were stimulated with BALF exosomes from 19 sarcoidosis patients. The asthma drug Montelukast (MK), a cysteinyl leukotriene receptor antagonist, was added 30 min before addition of exosomes from 15 ml of sarcoidosis patient BALF for 22 h. Levels of CCL2 in the cell-free supernatant were measured by a multiplex cytokine assay, and acquired by flow cytometry. n = 19 patients distributed on recipient cells from three different healthy volunteers.

The current study indicates that subgroups of patients have exosomes with far stronger capacity to induce pro-inflammatory cytokine release (Fig. 3). All stimulations were conducted on recipient cells from at least two separate healthy volunteers, and results were always reproducible in two or more cell donors. Our previous findings showed that BALF exosomes carry LT-forming enzymes, and induce LT-dependent pro-inflammatory effects^20,21^. We therefore investigated whether leukotrienes may be involved in the observed pro inflammatory effects. PBMCs were stimulated with BALF exosomes from 19 patients in the presence or absence of the CysLT1 receptor antagonist Montelukast. The CCL2 levels were not significantly different with Montelukast addition, but for 13 out of 19 patient exosome stimulations, Montelukast reduced CCL2 levels (Fig. 4). Further, six patient exosomes (out of 19) induced highly elevated CCL2 levels, and for all these six Montelukast had a potent inhibiting effect. PBS (exosome diluent) or Montelukast alone did not induce any increase in CCL2 (Fig. 4).

The CCL2 induction and Montelukast effect seemed most prominent in a subgroup of patients with more Lofgren`s syndrome (LS, an acute form of sarcoidosis) and possibly those receiving NSAID treatment. We therefore analysed a separate cohort with nine healthy controls and 30 patients evenly distributed in three groups; Non-LS patients, LS-patients with treatment, and LS-patients without treatment. PBMCs were stimulated with BALF exosomes for 22 h in presence or absence of Montelukast. Here, CCL-2 was again induced in a subgroup of patients, and Montelukast showed no overall significant impact on CCL2 levels, but a decrease in approximately half of patient exosomes (Supplementary Fig. 5). However no difference was seen between patients with non-LS, or LS patient with our without treatment (Supplementary Fig. 5).

### Sarcoidosis exosomes induce reactive oxygen species

**Figure 5:**
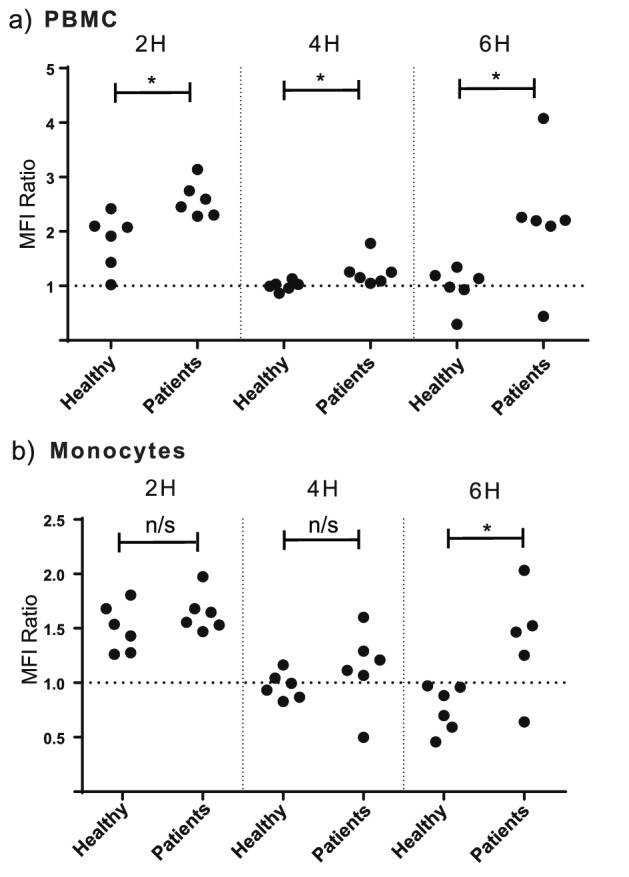
Exosomes from sarcoidosis BALF induce release of reactive oxygen species. PBMCs (**A**) or enriched monocytes (**B**) from healthy donors were stimulated with exosomes from 15 ml BALF of sarcoidosis patients or healthy volunteers for 2, 4 or 6 h. Released reactive oxygen species were measured using a kit for flow cytometric detection. The fluorescence was measured and normalised to that of PBS control (dotted line) according to manufacturer´s protocol, to produce a ratio of mean fluorescence intensity (MFI) for each stimulation. n = six vesicle donors distributed on two healthy recipient cell donors, *p < 0.05 using an non-paired T-test.

Monocytes generate ROS in inflammatory situations^17–19^, and we evaluated ROS production by PBMCs and enriched monocytes stimulated with sarcoidosis patient or healthy BALF exosomes. A statistically significant, but modest, increase in ROS was seen for patient exosome stimulated PBMC cultures at 2, 4 and 6 h, respectively whereas enriched monocytes displayed a small increase only at 6 h (Fig. 5).

## Discussion

Exosomes carry functional cargo in the form of proteins, nucleic acids and lipids, and we have previously reported that sarcoidosis BALF exosomes induce IFNγ in PBMCs and IL-8 in epithelial cells^4^, suggesting a role for sarcoidosis exosomes in T-cell activation and cell recruitment. We have in a proteomic characterisation also found enrichment in sarcoidosis exosomes for a large set of pro-inflammatory components, several of which have chemotactic properties on T cells but also on monocytes^5^. Many studies on sarcoidosis immunology focus on T cells, whereas we here aimed at further investigating sources of innate cytokines. Monocytes are potent cytokine producers^13^, and likely play a role in sarcoidosis^10–12^, and we focused our investigations on monocytes in parallel with whole PBMCs. During inflammation, monocytes are recruited to the affected tissue where they can contribute to inflammation both by differentiating to inflammatory DCs and macrophages^22^, but also by producing cytokines including TNF and IL-1β^23^. We and others argue for pathogenic roles of exosomes from the lungs, particularly in inflammatory conditions^6^. Whether exosomes can pass from the lung into systemic circulation, and there interact with circulating cells, remains to be proven but it is highly likely, based on the nature of exosomes to pass other barriers including the blood brain barrier^24^. The current study is based on interactions between pulmonary exosomes and MNPs, which can readily occur within the lungs, but we also hypothesise that these exosomes are able to reach systemic circulation, where they encounter blood monocytes, affect activation and potentially subsequent migration to the lungs.

We detected a clear induction of IL-1β in both enriched CD14^+^ monocytes, and in monocytes within PBMC populations, in a significant and dose-dependent manner by patient (but not healthy) exosomes (Fig. 2A,B). It should be noted that the CD14^+^ proportion in negatively enriched cultures reached around 75%, but our results suggest a direct effect of exosomes on the monocytes.

To expand cytokine analyses and to also investigate cytokine release from cells, we analysed supernatants of exosome-stimulated PBMCs or monocytes, using a multiplex assay, and found IL-1β, CCL2, TNF and IL-6 to be significantly induced by patient exosomes (Fig. 4A–D). IL1-β, TNF and IL-6 are all fundamentally associated with sarcoidosis, and CCL2 is a potent chemotactic agent for monocytes and T cells, and is strongly increased in BAL fluid of sarcoidosis patients^25,26^. Our findings are therefore in line with observed inflammatory activities in sarcoidosis, but expand mechanistic findings on the disease to include the exosomes/monocytes axis as a contributor to inflammation.

For each cytokine analysed, it is clear that patient exosomes did not induce normally distributed responses. Rather a group of patient exosomes induced much higher levels, which we argue is a result of the very complex nature of exosomes, but also great heterogeneity in sarcoidosis patients. In line with the observed complexity, CCL2 levels in BALF of sarcoidosis have previously been found strongly elevated but with a very large spread^26^. Whether “high inducer” patients in our current study represent a distinct clinical phenotype would be of great interest, but cross-referencing the results of the different cytokines to substantial patient data including age, sex, medication, disease status and lung function parameters did not reveal any clear correlations, other than that a few patients were efficient in inducing all cytokines. Also after expanding CCL2 analyses to a new cohort, no correlation was seen with these parameters (Supplementary Table 2 and Supplementary Fig. 4). We did observe that BALF exosomes from the four patients undergoing medication with non-steroidal anti-inflammatory drugs (NSAID) (Supplementary Table 1), all were amongst the high inducers for at least one cytokine (Fig. 3), however no clear correlation to NSAID treatment was seen in the new cohort (data not shown).

Leukotrienes are central in airway inflammation, and we have previously detected enrichment for leukotriene-forming enzymes in sarcoidosis exosomes^5,21^. Significant increases of Cysteinyl-leukotrienes (CysLTs) have also been found elevated in BAL fluid and exhaled breath concentrate of sarcoidosis patients^27,28^. However, to our knowledge, treating sarcoidosis with leukotriene inhibitors has not been widely considered. Only one trial has been conducted, where the CysLTR1 receptor antagonist Montelukast was tested in a small seven sarcoidosis patient cohort, and was found to inhibit TGFβ mRNA expression and fibrosis-favoring effects on myofibroblasts in vitro^29^. We previously demonstrated that asthma patient exosomes induce IL-8 in epithelial cells, which can be reduced by Montelukast^21^. We therefore set out in the current study to investigate if Montelukast also can modulate inflammatory effects induced by sarcoidosis exosomes. Monocytes, the cells in focus in this study, do express CysLTR1 and migrate in response to CysLT-activity^30^, so it is plausible that Montelukast would have an effect on monocytes. Further, CysLTs have been shown to induce the release of CCL2 by monocytes^31^, so we tested whether the CCL2 levels in sarcoidosis exosome-stimulations could be reduced by blocking CysLTR1. By adding Montelukast, the overall levels of CCL2 were not significantly changed (Fig. 4), but we did observe a decreased CCL2 level in 13 out of 19 patient exosome stimulations, and a strong reduction in CCL2 for the six patients inducing the highest levels.

Although Montelukast is broadly used as an asthma drug, it has been reported that only 42% of asthma patients respond with improved lung capacity^32^, in some cohorts the number is as low as 5%^33^, demonstrating that only a subgroup of asthma patients respond to the drug. Therefore, our observations that Montelukast can reduce CCL2 for at least a subgroup of sarcoidosis patients may have its explanation in a combination of heterogeneity in Montelukast efficacy, patient differences and the complex nature of exosomes. To have a robust assessment we therefore initiated a separate validation cohort of another 30 patients and 9 healthy controls and repeated the cytokine induction study and Montelukast inhibition assay. We found similar trends for CCL2 induction, and we again found that Montelukast had a heterogenous but interesting effect. For approximately half of the 30 sarcoidosis BALF exosome stimulations, the CCL2 levels were decreased by Montelukast (Supplementary Fig. 5). Paradoxically, approximately half of all stimulations in the new cohort also showed that the CCL2 levels were increased by MK addition, whereas MK alone (with no exosomes present) had no effect at all. It is unclear by what mechanisms this occurs, which speaks for further investigation of the complex mechanisms of exosomal leukotrienes.

There are minor differences in how the exosomes were isolated in the new cohort (see “Supplementary Methods”), so care should be taken if fusing the data, but if considered together, approximately 30 out of 50 patient exosome inductions of CCL2 were reduced by Montelukast. Although too early to suggest clinical studies, we find it very interesting that CCL2 levels could be reduced for a majority of patient exosome stimulations. Particularly so if considering the complexity and heterogeneity of exosomes derived from patient samples, and studies conducted on two cohorts.

Our data therefore provide grounds for further investigation of leukotriene-modulating drugs to modulate inflammatory mechanisms in sarcoidosis.

Monocytes produce ROS which are strongly pro inflammatory, and oxidative stress is implicated in the pathogenesis of lung disorders including sarcoidosis^16^. In the current study we found that PBMCs and enriched monocytes released significantly more ROS when stimulated by patient exosomes compared to healthy exosomes (Fig. 5). The observations support our other data, but as the effects are modest, experiments including more subjects would be needed to draw more definite conclusions on ROS production.

The cellular sources of BALF exosomes are still not clear, but they are likely a mixture of vesicles from epithelial cells and leukocytes, including activated dendritic cells, monocytes and T cells. Further studies will hopefully elucidate the relative functional contribution of exosomes of different cell sources, and contribute to the insights in inflammatory mechanisms in sarcoidosis.

In conclusion, this study supports a pro inflammatory effect of sarcoidosis exosomes on monocytes. The effects could at least partly be reduced by Montelukast, however further studies are needed to understand the mechanism of action and to elucidate whether the exosome-induced inflammation in sarcoidosis patients might be alleviated with anti-exosome or anti-leukotriene treatment. Considering their pro inflammatory nature, and repeated findings in the context of sarcoidosis, exosomes should also be further studied as biomarkers for treatment strategies in sarcoidosis.

## Materials and methods

### Patients and healthy volunteers

Patients diagnosed with sarcoidosis (n = 32, see Supplementary Table 1 for clinical data) and healthy volunteers gave informed consent for inclusion in this study, which was approved by the local ethics committee (Stockholm north ethical committee). All samples were handled in an anonymous, coded setup to protect integrity of participating patients and healthy volunteers. All experiments were performed in accordance with the Helsinki Declaration, local and national regulations, as well as guidelines and regulations of the Karolinska Institute and Karolinska Hospital, Sweden. Bronchoscopy with BAL was conducted as part of diagnostic routines at the Karolinska Hospital in Sweden. Sarcoidosis diagnoses were established according to World Association of Sarcoidosis and Other Granulomatous Disorders (WASOG) guidelines^34^ based on typical clinical signs, biopsy specimens showing non-caseating granuloma formation, and chest radiographic findings compatible with sarcoidosis. The diagnoses were further supported by differential BALF cell counts including BALF CD4/CD8 ratios and by ruling out other causes of these observations. Nine of the patients were diagnosed with Löfgren’s syndrome (LS) based on acute onset of the disease with fever, erythema nodosum, and/or ankle periarticular swelling and bilateral hilar lymphomas with or without concomitant parenchymal infiltrates. Due to limited sample access, LS and non-LS patients were not separated in this study. Healthy volunteers (n = 24, see Supplementary Table 1 for clinical data) with normal chest radiographs, blood cell counts, and electrolytes provided informed consent to undergo BAL, and for their specimens to be used in research. The expanded validation cohort of 30 patients and 9 healthy controls was recruited and handled under similar conditions, see Supplementary Table 2.

### Exosome isolations

Exosomes were isolated as described previously^4,5^. Immediately after collection, the BAL fluid was cooled on ice until processed by centrifugation, pelleting cells for 10 min at 400*g*. Shortly thereafter, 50–150 ml BAL fluid per subject was further processed to pellet cell debris by 3,000 × *g* centrifugations for 40 min. The BAL fluid supernatant was then frozen at − 80 °C and kept until thawn in + 4 °C overnight for further EV isolations. After thawing, the BAL fluid was centrifugated at 10,000 × *g* for 40 min and filtered through 0.22 μM filter to remove debris and larger vesicles. Finally, exosomes were isolated by 140,000 × *g* centrifugation for 2H, and pellets were washed in large volumes of PBS. The exosomes were resuspended in PBS volumes normalised to the volume of original BAL fluid, and kept at − 80 °C until use. All high speed centrifugations (10,000 × *g* and above) were conducted using a fixed-angle rotor (Ti45, Beckman-Coulter), and g numbers represent RCFmax with a low brake. Of note, free soluble proteins including cytokines generally require centrifugation at higher speed (several 100,000 × *g*) to be pelleted, and are therefore not present in exosome pellets in relevant amounts.

### Exosome characterisations

Exosomes from healthy controls, as well as sarcoidosis patients, isolated using identical protocols and the same settings, have been thoroughly characterised for morphology, total protein amounts, full proteomic content and non-vesicular contaminations in our previous publications^4,5^, where we verified the vesicular nature of these exosomes fulfilling the criteria of the MISEV 2018 guidelines^35^. Exosomes were quantified and analysed using a Nanoparticle Tracking Analysis (NTA) instrument, NanoSight LS14 (Malvern). Samples were diluted in PBS to achieve around 40 particles per frame, and a concentration between 1 and 10 × 10^8^ particles per ml. Five 30 s videos of each sample were recorded under continuous flow by a syringe pump, using camera level 10. Batch analyses were made using threshold level 3, to analyse exosome sizes, and for calculations of concentrations of exosomes in the original BAL fluids.

### Cell isolations

Buffy coats from healthy blood donors were acquired from the hospital after informed consent for their specimens to be used in research. The buffy coats were divided for isolations of peripheral blood mononuclear cells (PBMC) by Ficoll density based separation, and monocytes by negative selection using the Rosettesep (Stemcell) antibody-based depletion cocktail combined with density-based separation. Residing erythrocytes were lysed using ACK buffer for 90 s followed by extensive washing of the leukocytes. Cells were counted and viabilities were determined using trypan blue.

### Cell stimulations for intracellular cytokine analysis

For the flow cytometric analysis experiments, 500,000 PBMCs or 250,000 monocytes were resuspended in Aim V serum-free media (Gibco) and were added to 48-well plates. The cells were then stimulated with exosomes from healthy donors and sarcoidosis patients isolated from 1, 5 and 15 ml of BALF. As reported in Fig. 1C, these BALF volumes typically correspond to 7.3; 36.5; and 146 × 10^6^ vesicles/well for patients. Negative control was PBS added in equal volume as the PBS-suspended vesicles, positive controls were PMA/Ionomycin cocktail used at 1× dilution (Biolegend), Concanavalin A or 100 ng/ml LPS. To block cytokine release for the intracellular stainings, Monensin (Biolegend) used at 1× dilution according to manufacturer’s protocol was added after 2 h of stimulation. After 6 h of stimulation, plates were spun down at 350 × *g* for 10 min and cells were resuspended in ice cold PBS + 0.5 mM EDTA and incubated on ice for 5 min to detach cells, before transferring them to 96 well plates for analysis.

### Cell stimulations for analysis of released cytokines

PBMCs and enriched monocytes were resuspended in Aim V serum-free media (Gibco) and 180,000 cells per well were added to 96 well plates. The cells were then stimulated with exosomes from healthy donors or sarcoidosis patients isolated from 5 ml of BALF, to have equal numbers of EVs per cell as in the intracellular cytokine analysis described above. As reported in Fig. 1C, 5 ml of BALF typically correspond to 37 × 10^6^ (for patients) and 14 × 10^6^ (for healthy controls) vesicles. Negative control was PBS added in equal volume as the PBS-suspended vesicles. Blocking experiments with the CysLT-receptor antagonist Montelukast were identically performed, and with a 30 min pre-incubation of the cells with a 1.1 μM concentration of Montelukast, which is comparable to the blood concentration of Montelukast in a patient on normal dosage. After 6 or 22 h respectively, the cells were spun down by 350 g for 10 min before the supernatants were transferred to new plates and frozen immediately at − 80 °C for later analysis.

### Flow cytometric analyses

Surface proteins and intracellular cytokines were detected by flow cytometry to analyse the impact of pulmonary EVs on T cells and NK cells in one panel, monocytes and dendritic cells in a second panel. All cells were washed in PBS, followed by FC blocking and surface staining in 96 well plates. Permeabilisation, fixation and intracellular staining was performed according to the manufacturer´s protocol (BD Biosciences). Cells were stained for surface HLA-DR, CD14, CD16, (for T/NK cells also CD3, CD56, CD4 and CD8) and intracellular IFNγ, and IL-1β. Lineage markers included in the monocyte gatings were CD3, CD19, CD56 and CD66b. For T/NK cell panels the lineage markers used were CD19, CD11c, CD14, and CD66b. The cells were acquired in an LSR Fortessa flow cytometer (BD Biosciences), and the results were gated using FlowJo software (LLC Inc). Result plots and statistical analyses were conducted in GraphPad (Prism Software).

### Multiplex cytokine analysis

For analysis of supernatants of stimulated cells, cytometric bead array (CBA) was conducted according to the manufacturer’s protocol (Becton–Dickinson), for IL-1β, IL-6, IFNγ, TNF, and CCL2. The samples were acquired on an LSR Fortessa flow cytometer (Becton–Dickinson) and analysed in FCAP Array software (Becton–Dickinson).

### Reactive oxygen species

Reactive oxygen species were analysed in a flow cytometer-based kit according to the manufacturer´s protocol (Abcam). PBMCs or enriched monocytes (150,000 per well) were stimulated with exosomes corresponding to 15 ml BALF from patients or healthy controls for 2, 4 or 6 h. Samples were acquired using a LSR Fortessa flow cytometer (Becton–Dickinson), gated using FlowJo software (Becton–Dickinson/LLC Inc) and plotted as differences in MFI ratio to PBS-stimulated control according to manufacturers’ recommendation.

## Supplementary information

Supplementary Information.

## References

[1] Patterson KC, Strek ME (2013). Pulmonary fibrosis in sarcoidosis. Clinical features and outcomes. Ann. Am. Thorac. Soc..

[2] Baughman RP, Culver DA, Judson MA (2011). A concise review of pulmonary sarcoidosis. Am. J. Respir. Crit. Care Med..

[3] Cicero AL, Stahl PD, Raposo G (2015). Extracellular vesicles shuffling intercellular messages: For good or for bad. Curr. Opin. Cell Biol..

[4] Qazi KR (2010). Proinflammatory exosomes in bronchoalveolar lavage fluid of patients with sarcoidosis. Thorax.

[5] Martinez-Bravo MJ (2017). Pulmonary sarcoidosis is associated with exosomal vitamin D-binding protein and inflammatory molecules. J. Allergy Clin. Immunol..

[6] Wahlund CJE, Eklund A, Grunewald J, Gabrielsson S (2017). Pulmonary extracellular vesicles as mediators of local and systemic inflammation. Front. Cell Dev. Biol..

[7] Baharom F, Rankin G, Blomberg A, Smed-Sörensen A (2017). Human lung mononuclear phagocytes in health and disease. Front. Immunol..

[8] Lepzien R (2019). Mapping mononuclear phagocytes in blood, lungs, and lymph nodes of sarcoidosis patients. J. Leukoc. Biol..

[9] Grunewald J, Spagnolo P, Wahlström J, Eklund A (2015). Immunogenetics of disease-causing inflammation in sarcoidosis. Clin. Rev. Allergy Immunol..

[10] Sakthivel P, Grunewald J, Eklund A, Bruder D, Wahlstrom J (2016). Pulmonary sarcoidosis is associated with high-level inducible co-stimulator (ICOS) expression on lung regulatory T cells-possible implications for the ICOS/ICOS-ligand axis in disease course and resolution. Clin. Exp. Immunol..

[11] Fraser SD, Sadofsky LR, Kaye PM, Hart SP (2016). Reduced expression of monocyte CD200R is associated with enhanced proinflammatory cytokine production in sarcoidosis. Sci. Rep..

[12] Okamoto H, Mizuno K, Horio T (2003). Circulating CD14+ CD16+ monocytes are expanded in sarcoidosis patients. J. Dermatol..

[13] Iwasaki A, Foxman EF, Molony RD (2017). Early local immune defences in the respiratory tract. Nat. Rev. Immunol..

[14] Crommelin HA (2014). Anti-TNF therapeutics for the treatment of sarcoidosis. Immunotherapy.

[15] Hijdra D (2016). Can intermediate monocytes predict response to infliximab therapy in sarcoidosis?. Eur. Respir. J..

[16] Bargagli E (2009). Oxidative stress in the pathogenesis of diffuse lung diseases: A review. Respir. Med..

[17] Watanabe T, Yasunari K, Nakamura M, Maeda K (2006). Carotid artery intima-media thickness and reactive oxygen species formation by monocytes in hypertensive patients. J. Hum. Hypertens..

[18] Himmelfarb J, Lazarus JM, Hakim R (1991). Reactive oxygen species production by monocytes and polymorphonuclear leukocytes during dialysis. Am. J. Kidney Dis..

[19] Dubey ML (1991). Generation of reactive oxygen species by blood monocytes in human *Plasmodium falciparum* and *P. vivax* infections. Apmis.

[20] Esser J (2010). Exosomes from human macrophages and dendritic cells contain enzymes for leukotriene biosynthesis and promote granulocyte migration. J. Allergy Clin. Immunol..

[21] Paredes PT (2012). Bronchoalveolar lavage fluid exosomes contribute to cytokine and leukotriene production in allergic asthma. Allergy.

[22] Shi C, Pamer EG (2011). Monocyte recruitment during infection and inflammation. Nat. Rev. Immunol..

[23] Yang J, Zhang L, Yu C, Yang XF, Wang H (2014). Monocyte and macrophage differentiation: Circulation inflammatory monocyte as biomarker for inflammatory diseases. Biomarker Res..

[24] Saint-Pol J, Gosselet F, Duban-Deweer S, Pottiez G, Karamanos Y (2020). Targeting and crossing the blood-brain barrier with extracellular vesicles. Cells.

[25] Palchevskiy V (2011). Immune response CC chemokines CCL2 and CCL5 are associated with pulmonary sarcoidosis. Fibrogenesis Tissue Repair.

[26] Hamsten C (2016). Elevated levels of FN1 and CCL2 in bronchoalveolar lavage fluid from sarcoidosis patients. Respir. Res..

[27] Piotrowski WJ (2007). Eicosanoids in exhaled breath condensate and BAL fluid of patients with sarcoidosis. Chest.

[28] Antczak A (2011). Correlation between eicosanoids in bronchoalveolar lavage fluid and in exhaled breath condensate. Dis. Markers.

[29] Fireman E, Schwartz Y, Mann A, Greif J (2004). Effect of montelukast, a cysteinyl receptor antagonist, on myofibroblasts in interstitial lung disease. J. Clin. Immunol..

[30] Woszczek G (2008). Leukotriene D(4) induces gene expression in human monocytes through cysteinyl leukotriene type I receptor. J. Allergy Clin. Immunol..

[31] Ichiyama T (2005). Cysteinyl leukotrienes induce monocyte chemoattractant protein 1 in human monocytes/macrophages. Clin. Exp. Allergy J. Br. Soc. Allergy Clin. Immunol..

[32] Malmstrom K (1999). Oral montelukast, inhaled beclomethasone, and placebo for chronic asthma. A randomized, controlled trial. Montelukast/Beclomethasone Study Group. Ann. Internal Med..

[33] Szefler SJ (2005). Characterization of within-subject responses to fluticasone and montelukast in childhood asthma. J. Allergy Clin. Immunol..

[34] Hunninghake GW (1999). ATS/ERS/WASOG statement on sarcoidosis. American Thoracic Society/European Respiratory Society/World Association of Sarcoidosis and other Granulomatous Disorders. Sarcoidosis Vasculitis Diffuse Lung Diseases Off J. WASOG/World Assoc. Sarcoidosis Other Granulomatous Disord..

[35] Thery C (2018). Minimal information for studies of extracellular vesicles 2018 (MISEV2018): A position statement of the International Society for Extracellular Vesicles and update of the MISEV2014 guidelines. J. Extracell Vesicles.

