# Supplementary material for: Sarcoidosis exosomes stimulate monocytes to produce pro-inflammatory cytokines and CCL2
Source: Sci Rep. 2020 Sep 18;10:15328. doi: 10.1038/s41598-020-72067-7 (PMC7501276; doi:10.1038/s41598-020-72067-7)
Supplement: Supplementary file 1 — Supplementary Information. [file 41598_2020_72067_MOESM1_ESM.pdf]

Supplementary information

Sarcoidosis exosomes stimulate monocytes to produce pro-inflammatory cytokines and CCL2

[1,2]Casper JE Wahlund, [1,2]Gozde Gucluler Akpinar, [1,2]Loïc Steiner, [1,2]Ahmed Ibrahim, [1,2]Elga Bandeira,[1,2]Rico Lepzien, [5]Ana Lukic, [1,2]Anna Smed-Sörensen, [3,4]Susanna Kullberg, [3,4]Anders Eklund, [3,4]Johan Grunewald, [1,2]Susanne Gabrielsson

1 Karolinska Institutet, Department of Medicine Solna, Division of Immunology and Allergy

2 Karolinska University Hospital, Department of Clinical Immunology and Transfusion Medicine

3 Karolinska Institutet, Department of Medicine Solna, Respiratory Medicine Division

4 Karolinska University Hospital, Department of Respiratory Medicine

5 Karolinska Institutet, Department of Clinical Neuroscience, Therapeutic immune design

Correspondence to Susanne.gabrielsson@ki.se

Supplementary figure 1a

PBMCs

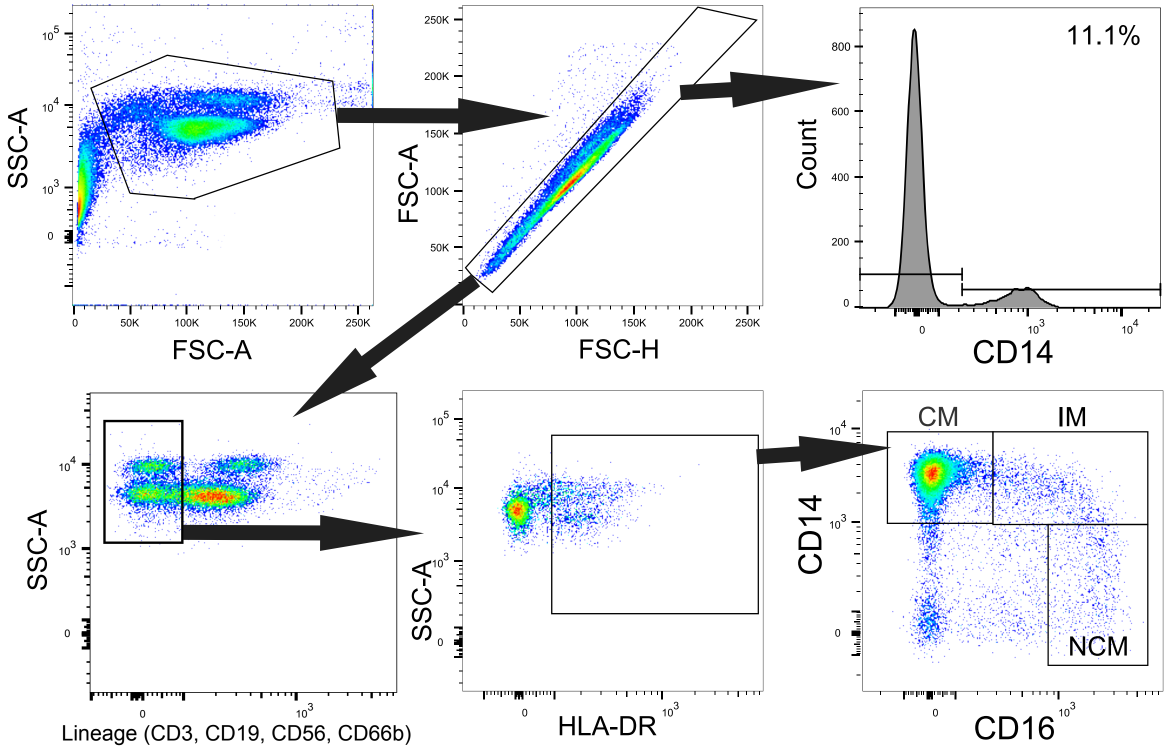

Enriched monocytes

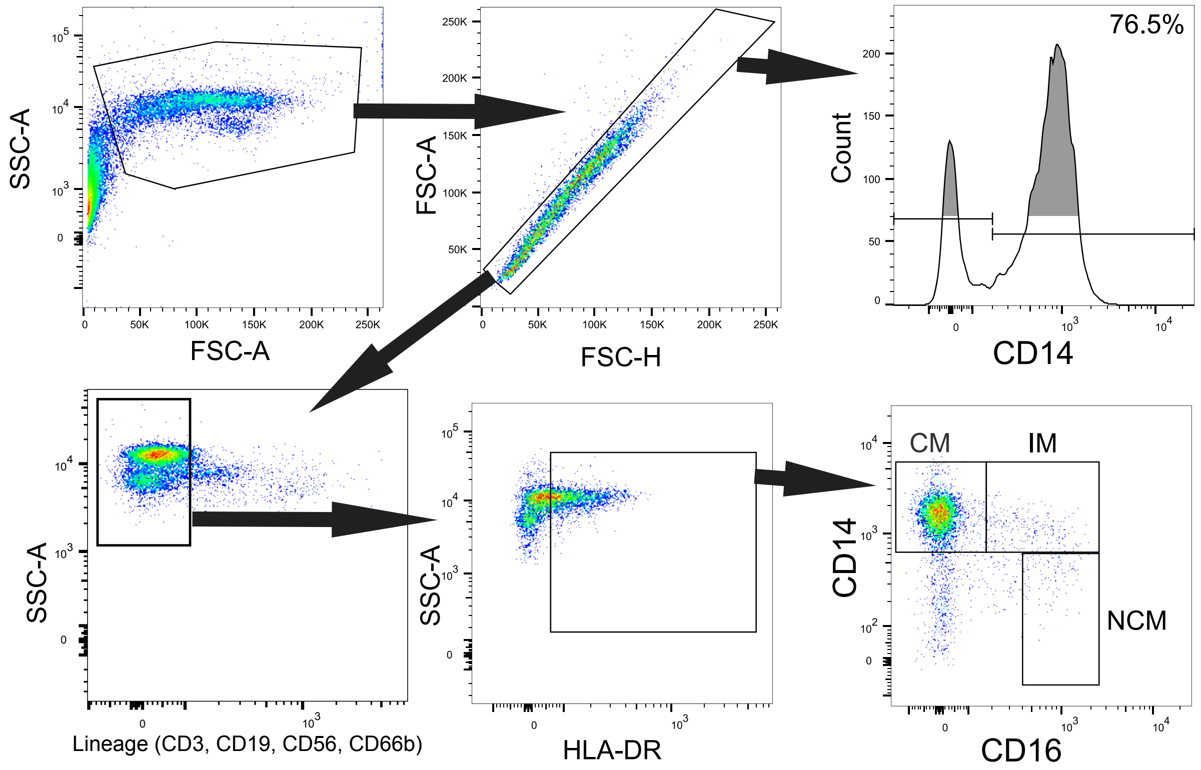

Supplementary figure 1b

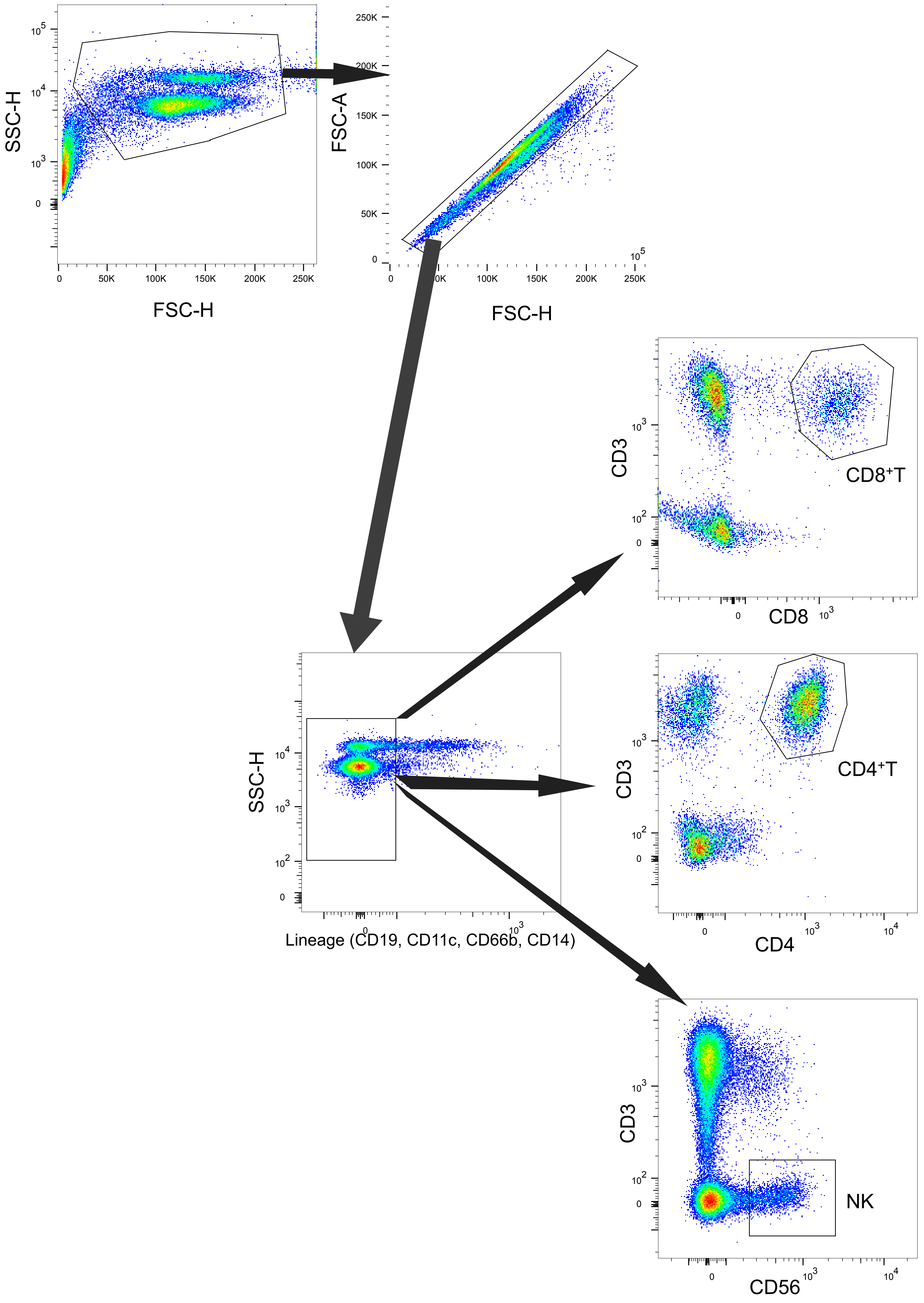

Supplementary figure 2

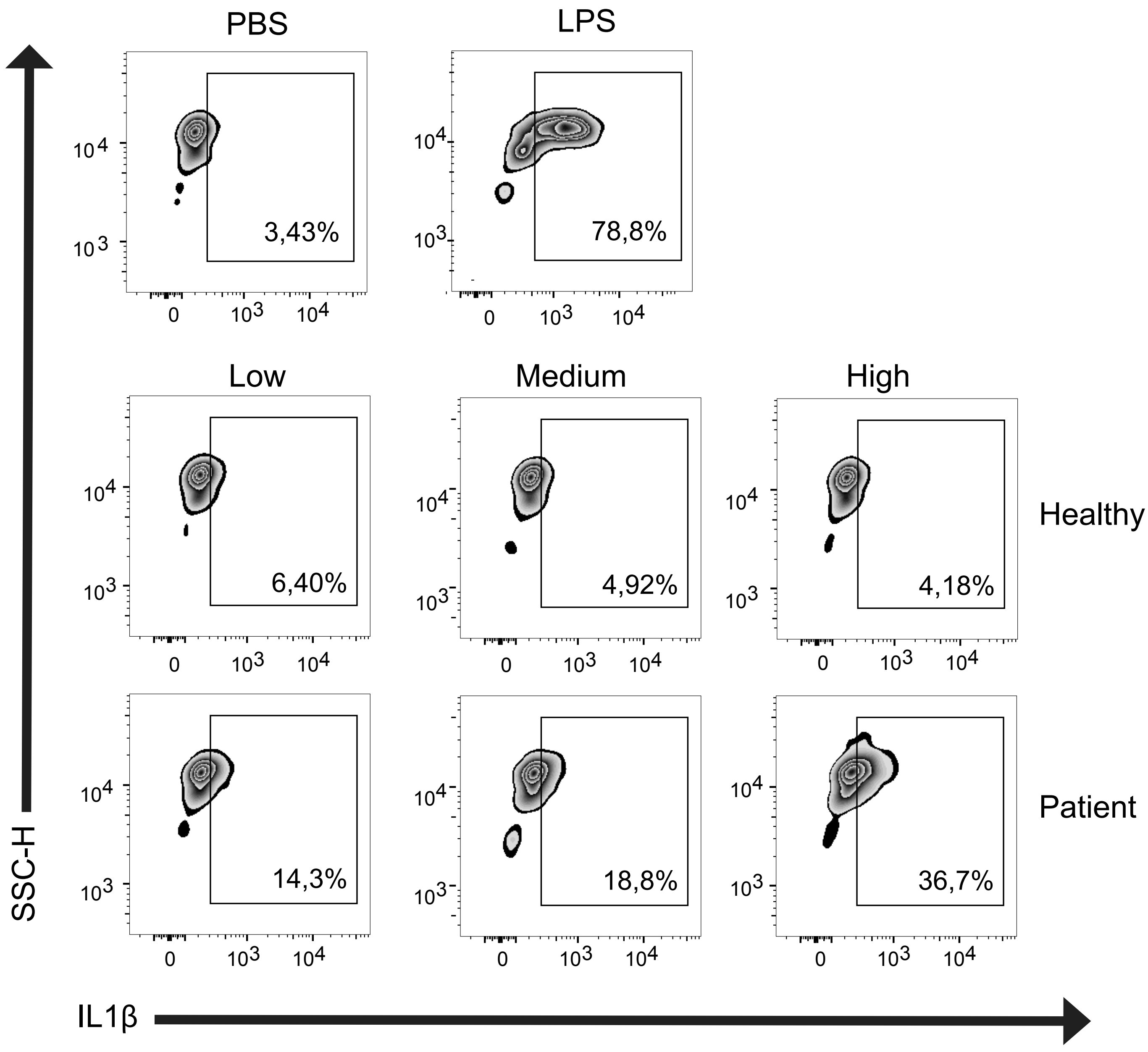

Supplementary figure 3

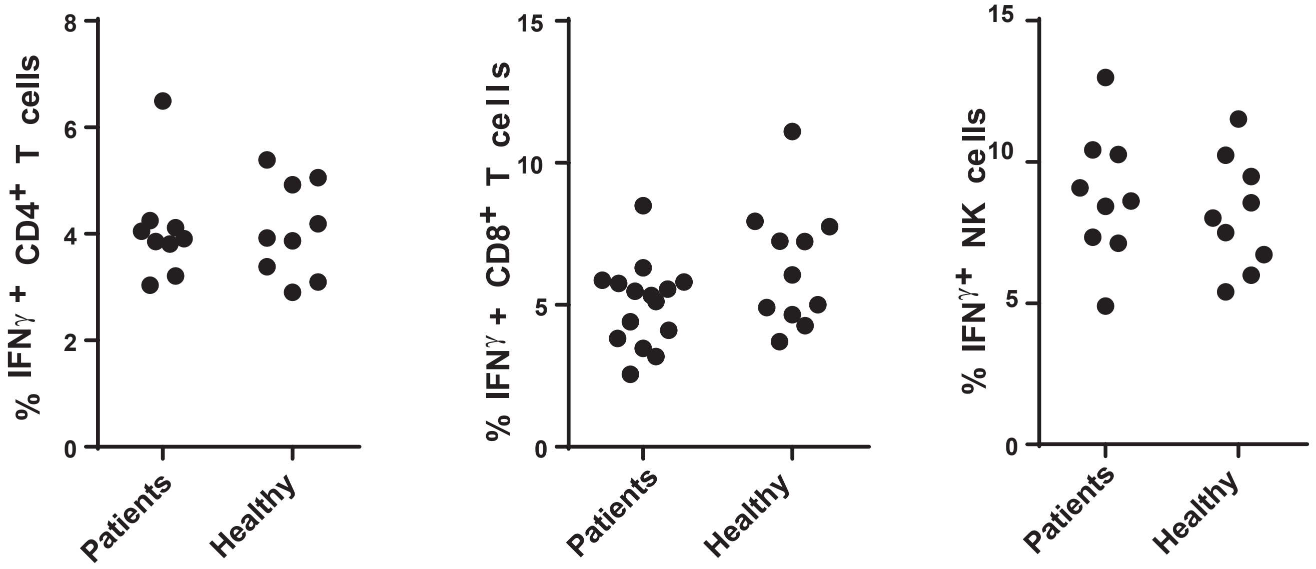

Supplementary figure 4

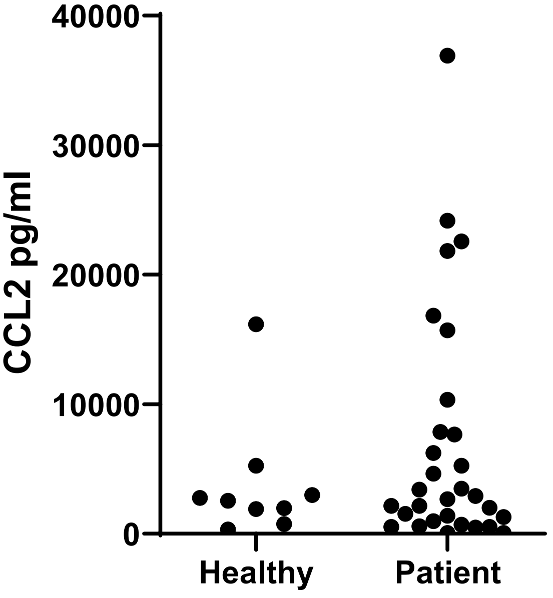

Supplementary figure 5

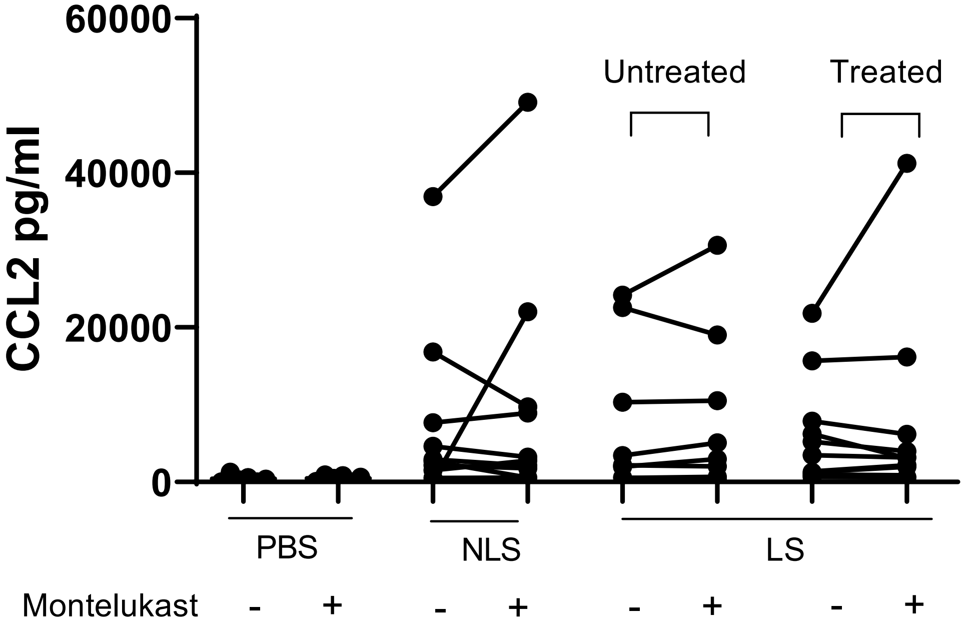

Supplementary table 1

Patients

Symbol	Age	Sex	Löfgren's	Smoke	Stage	Medication
○ (black)	35	F	N	Y	1	
◇ (black)	36	F	N	N	-	
○ (blue)	36	F	N	Y	-	
△ (black)	47	F	N	N	2	
□ (black)	49	F	N	N	1	
☒ (black)	50	F	N	N	2	
□ (blue)	50	F	N	N	-	
⊗ (black)	52	F	N	Y	-	Paracetamol
● (blue)	59	F	N	N	2	
▼ (blue)	59	F	N	Ex	2	
▽ (black)	65	F	N	N	-	
● (red)	22	F	Y	N	1	
◇ (red)	27	F	Y	Y	1	
▲ (red)	36	F	Y	Y	1	
△ (red)	40	F	Y	N	2	NSAID
□ (red)	55	F	Y	N	2	NSAID, Inh corticoster
◆ (blue)	28	M	N	Ex	2	Inh Corticoster
■ (black)	31	M	N	N	2	
◆ (black)	33	M	N	N	2	
✖ (black)	33	M	N	N	2	Inh corticoster
▲ (blue)	36	M	N	Ex	1	Inh corticoster
✖ (blue)	39	M	N	N	1	
△ (blue)	41	M	N	Y		
○ (red)	42	M	N	Ex	2	
⊗ (red)	43	M	N	Ex	2	Inh corticoster
▼ (black)	44	M	N	N	2	Inh corticoster
■ (blue)	47	M	N	Ex	1	
⊗ (blue)	48	M	N	N	1	
▼ (red)	33	M	Y	Y	1	NSAID
■ (red)	40	M	Y	N	2	
◆ (red)	46	M	Y	Ex	1	
✖ (red)	51	M	Y	N	1	Paracetamol + NSAID

Healthy controls

Age	Sex	Smoke
51	F	N
21	F	N
20	F	N
19	F	N
25	F	Y
52	F	N
26	F	N
57	F	N
27	F	Ex
22	F	N
35	F	N
43	M	N
20	M	N
35	M	N
24	M	N
37	M	N
29	M	Y
22	M	Y
25	M	Y
23	M	N
19	M	N
28	M	N
29	M	N
29	M	N
26	M	N

Supplementary table 2

Age	Sex	Löfgren	Smoke	Medication
46	M	N	N	
26	M	N	N	
35	F	N	N	
62	M	N	N	
58	M	N	N	
56	M	N	N	
58	M	N	N	
43	M	N	N	
50	M	N	N	
65	M	N	N	
48	F	Y	N	
41	M	Y	N	NSAID
43	M	Y	N	Corticosteroids + NSAID
59	M	Y	N	
36	M	Y	N	NSAID
49	M	Y	N	Paracetamol + NSAID
62	M	Y	N	
51	M	Y	N	
31	M	Y	N	NSAID
36	M	Y	N	Paracetamol + NSAID
47	M	Y	N	
36	F	Y	N	
37	M	Y	N	NSAID
45	M	Y	N	Cortisone + NSAID
42	M	Y	N	
46	M	N	N	
38	F	N	N	
39	M	Y	N	NSAID
42	M	Y	N	
50	F	Y	N	

Figure legends

Supplementary figure 1

Gating strategy used in intracellular cytokine staining (Fig. 2). A. Classical monocytes were gated from PBMCs or enriched monocytes as $CD14^{+}CD16^{-}$ cells out of all single, lineage negative (CD3, CD56, CD66b, CD19), $HLA\text{-}DR^{Hi}$ cells. The proportion of CD14+ cells amongst the single cells were plotted as test of monocyte enrichment purity, showing around 75% enrichment.

B. T and NK cells were gated for single, lineage negative (CD19, CD11c, CD14, CD66b) cells followed by $CD3^{+}CD4^{+}$, $CD3^{+}CD8^{+}$ or $CD3^{-}CD56^{+}$.

Supplementary figure 2

Typical results for intracellular staining of IL1-β positive classical monocytes. PBMCs or enriched monocytes stimulated with EVs isolated from BAL fluid of sarcoidosis patients or healthy controls for six hours were analysed by intracellular flow cytometry. Numbers represent percent positive classical monocytes, gated as in supplementary Fig. 1A. A representative healthy donor and patient is displayed for three different exosome doses representing: low; 1ml, medium; 5ml, and high; 20ml of BALF/250 000 cells. PBS and LPS (100 ng/ml) were used as controls.

Supplementary figure 3

Intracellular staining of IFN-γ after exposure to allogeneic exosomes. PBMCs isolated from healthy donors were stimulated for six hours with exosomes from 20 ml BAL fluid of patients or healthy volunteers. An equivalent volume of PBS was used as negative control (data not shown). CD4+ T cells, CD8+T cells and NK cells were gated as in supplementary Fig. 1B. No elevation of $IFN\gamma^{+}$ cells were found. n=9-15 subjects per group, divided on at least three different healthy donor cell recipients.

Supplementary table 1

List of patients (n=32) and healthy volunteers (n=25) included in the study. Patients were 50% male/female ratio (n=16 each), with an age distribution of 22-65 years (average 42.3), nine diagnosed with Löfgren´s syndrome, and disease staging where known was 1 (n=11) or 2 (n=14). Smokers and ex-smokers represented 22% respectively (n=7), non-smokers 56% (n=18). Healthy volunteers were 44% females (n=11), and 56% males with the age distribution 19-51 years (average 28.7), 16% smokers (n=4), 4% ex-smokers (n=1) and 80% non-smokers (n=20).

Separate validation cohort data

Supplementary figure 4

PBMCs from healthy donors were stimulated for 22 hours with exosomes from 5 ml BALF from sarcoidosis patients or healthy donors. PBS was used as negative control. A multiplex cytokine assay acquired by flow cytometry was used to analyse concentrations of CCL2 in supernatants.

n = 30 patient exosomes and 9 healthy volunteer exosomes distributed on at least three healthy recipient cell donors.

Supplementary figure 5

Healthy donor PBMCs were stimulated with BALF exosomes from sarcoidosis patients (n=30). The asthma drug Montelukast (MK), a cysteinyl leukotriene receptor antagonist, was added 30 min before addition of exosomes from 5 ml of sarcoidosis patient BALF for 22 hours. Levels of CCL2 in the cell-free supernatant were measured by a multiplex cytokine assay, and acquired by flow cytometry.

Supplementary table 2

List of patients (n=30) included in the separate validation cohort. Patients were 83% male, with an age distribution of 26-65 years, 18 diagnosed with Löfgren´s syndrome, and medication status. All were non-smokers.

Supplementary methods - exosome isolations

Exosome isolations were conducted nearly identical to those of the main cohort, with the difference that the BAL fluid was frozen after pelleting cells (at 400g for 10 min). For exosome isolations, the BAL fluids were thawn and further processed in the same manner as for the main cohort; 3.000g centrifugations for 40 min, then 10.000g centrifugation of the supernatant for 40 min followed by 0.22 micrometer filtrations, and last 140.000g centrifugation for 2H. The exosomes were resuspended in PBS volumes normalised to the volume of original BAL fluid, and kept at -80°C until use.
